# Opioids and Immunosuppression: Clinical Evidence, Mechanisms of Action, and Potential Therapies

**DOI:** 10.1089/pmr.2023.0049

**Published:** 2024-02-02

**Authors:** Jeffrey J. Bettinger, Bruce C. Friedman

**Affiliations:** ^1^ Pain Management, Saratoga Hospital Medical Group, Saratoga Springs, New York, USA.; ^2^ JM Still Burn Center, Doctors Hospital of Augusta, Augusta, Georgia, USA.

**Keywords:** constipation, immunosuppression, laxative, methylnaltrexone, opioid-induced, PAMORA, peripheral μ-opioid receptor antagonists

## Abstract

**Background:**

In addition to the more well-known adverse effects of opioids, such as constipation, mounting evidence supports underlying immunosuppressive effects as well.

**Methods:**

In this study, we provide a narrative review of preclinical and clinical evidence of opioid suppression of the immune system as well as possible considerations for therapies.

**Results:**

*In vitro* and animal studies have shown clear effects of opioids on inflammatory cytokine expression, immune cell activity, and pathogen susceptibility. Observational data in humans have so far supported preclinical findings, with multiple reports of increased rates of infections in various settings of opioid use. However, the extent to which this risk is due to the impact of opioids on the immune system compared with other risk factors associated with opioid use remains uncertain. Considering the data showing immunosuppression and increased risk of infection with opioid use, measures are needed to mitigate this risk in patients who require ongoing treatment with opioids. In preclinical studies, administration of opioid receptor antagonists blocked the immunomodulatory effects of opioids.

**Conclusions:**

As selective antagonists of peripheral opioid receptors, peripherally acting mu-opioid receptor (MOR) antagonists may be able to protect against immune impairment while still allowing for opioid analgesia. Future research is warranted to further investigate the relationship between opioids and infection risk as well as the potential application of peripherally acting MOR antagonists to counteract these risks.

## Introduction

Opioids remain an integral part of a multimodal pain management approach for the treatment of moderate and severe pain in malignant and nonmalignant chronic conditions, as well as in perioperative and postoperative settings.^1–5^ Opioids exert their analgesic effects by binding to seven transmembrane-spanning G protein-coupled receptors (GPCRs). GPCRs comprise classical and nonclassical receptors; classical GPCRs include three main subtypes: mu, kappa, and delta.^6^

Although opioids can elicit pharmacological effects through kappa and delta opioid receptors, most opioids act primarily at mu-opioid receptors (MORs), which is their primary mode of mediating analgesia.^7^ Opioid binding to and activation of MORs causes inhibition of the release of excitatory neurotransmitters from presynaptic neurons while hyperpolarizing postsynaptic neurons.^7,8^

Opioid receptors are present in the central nervous system and the periphery, such as the gastrointestinal tract and the heart, in both neuronal and non-neuronal tissues.^7^ Analgesia is primarily mediated by opioid receptors in the CNS, although there is evidence that receptors in the peripheral nervous system can mediate analgesia as well.^9^ Opioid receptors in the enteric nervous system mediate gastrointestinal motility, gastrointestinal secretion, and electrolyte balance.^10–12^

Opioid receptors are also expressed on a variety of cell types, such as neurons, neutrophils, macrophages, and T and B cells.^8^ Because opioid receptors are widely distributed throughout the body, their activation can be associated with a variety of adverse effects, including sedation, constipation, tolerance, nausea, itch (pruritus), physical dependence, vomiting, dizziness, respiratory depression, hyperalgesia, and vasodilation and hypotension due to histamine release.^13,14^

In addition to the well-known side effects of opioids, opioids may be associated with another less recognized potential adverse effect: immunosuppression and potential increased risk for infection.^13,15^ Given the continued clinical use of opioid analgesics, the potential largely unexplored risk of opioid-related infections is a topic of concern.^15^ The aim of this narrative review is to discuss potential mechanisms that may contribute to immunosuppression, summarize the current evidence from clinical studies on the immunosuppressive effects of opioids and consequent outcomes across varied patient populations, and review recent data on use of opioid receptor antagonists for ameliorating immunosuppression.

## Pathophysiology of Opioid Effects on the Immune System

There is an increasing body of preclinical evidence on the immunosuppressive properties of exogenous opioids, predominantly morphine. The effects of endogenous opioids on the immune system are complex and can be either immunostimulatory or inhibitory, depending on factors such as cell type, based on *in vitro* studies.^8,16^

**FIG. 1.**
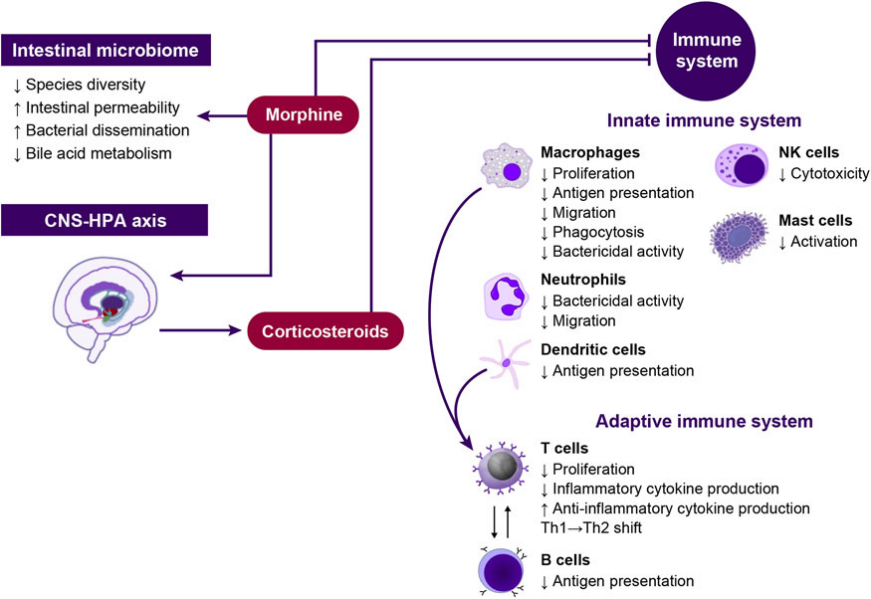
Proposed mechanisms of opioid-mediated immunosuppression.^8,17,18^ CNS, central nervous system; HPA, hypothalamic–pituitary–adrenal; NK, natural killer; Th, helper T cell.

The immune system comprises innate and adaptive systems. Preclinical studies have demonstrated that opioid use compromises both innate and adaptive immunity (Fig. 1).^8,17,18^ Opioids can alter the immune system through direct and indirect pathways. Direct pathways involve interaction of opioids with receptors on immune cells.^8,16^ Indirect pathways, however, may occur through opioid activation within the CNS and the hypothalamic pituitary–adrenal axis, resulting in release of corticosteroids and immunosuppressive hormones.^8,16^ The effect of opioids on innate and adaptive immune systems has been reviewed in detail and thus will be briefly summarized here.^8,17–19^

### Effect of opioids on innate immunity

Morphine has been shown to activate MORs on a variety of innate immune cells.^17–19^ In macrophages, this results in reduced proliferative capacity, inhibited recruitment, impaired phagocytosis, and reduced bactericidal activity.^17–19^ Although mechanistic details are not fully understood, these immunomodulatory effects appear to be mediated in part by crosstalk with Toll-like receptors (TLR) and altered nuclear factor-kappa B (NF-κB) signaling.^17,18^

Morphine inhibits the migratory activity of neutrophils through impairment of interleukin (IL)-8 signaling and reduces neutrophil superoxide production, impairing bactericidal activity.^17,18^ In addition, morphine blocks antigen presentation by dendritic cells through inhibition of IL-23 production. Morphine reduces natural killer (NK) cell cytotoxicity indirectly as a result of MOR activation in the CNS. Furthermore, morphine reduces mast cell activation, which increases intestinal permeability, and consequently, increases the risk of infection.^17,18^

### Effect of opioids on adaptive immunity

Opioid effects on the adaptive immune system appear to be more complex and, in some instances, less direct. For example, morphine decreases major histocompatibility complex class II expression on B cells, resulting in reduced antigen presentation and thus prohibiting activation of T cells.^17^ It is not certain whether this is mediated directly by MOR activation on immune cells or indirectly. Some evidence has suggested this effect is mediated by activation of the hypothalamic–pituitary–adrenal axis and consequent corticosteroid production.^20^

With regard to T cells, morphine reduces cell viability, proliferative response, T-helper cell function, and CD4/CD8 population.^8^ These effects are also observed *in vitro*, indicating MORs are directly activated on immune cells. In addition, morphine reduces production of IL-1β, IL-2, tumor necrosis factor-alpha, and interferon-gamma and stimulates production of the anti-inflammatory cytokines transforming growth factor-beta 1 and IL-10.^8^

Furthermore, morphine results in T-helper cell differentiation toward the Th2 lineage.^8^ As with the innate immune system, modulation of the NF-κB transcriptional pathway appears to be central to these effects.^21^ Literature on the effects of morphine on B cells is more limited. Morphine reduces B cell proliferation and antibody production. Although this is likely due to lack of stimulation from macrophages and T cells rather than a direct effect on B cells, there is evidence from *in vitro* studies that morphine may directly inhibit the humoral immune response as well.^8,17,22^

### Effect of opioids on the microbiome

A balanced gut microbiome and mucosal barrier function are central to effectively defending against harmful pathogens.^23^ A properly balanced gut microbiome protects against colonization by producing antimicrobial products (including short-chain fatty acids, bile acids, and bacteriocins), competing for nutrients, and maintaining epithelial barrier integrity. In addition to protection against enteric pathogens, it has been shown that the gut microbiome influences the vaccine-induced systemic immune response.^24^

Preclinical studies suggest that use of morphine disrupts the gut microbiome by decreasing the number of bacterial species, increasing intestinal permeability and bacterial translocation to mesenteric lymph nodes and the liver, and dysregulating immune responses and bile acid metabolism (Fig. 1).^25–28^ Clinical studies have echoed results from preclinical studies. An analysis of the microbiome of patients being treated for opioid use disorder showed lower diversity among patients taking opioid agonists (including heroin and prescription opioids) compared with patients not taking opioid agonists.^29^

A different study involving patients with cirrhosis showed that chronic opioid exposure was associated with reduced levels of beneficial short-chain fatty acid–producing bacteria in the patients' microbiome, worse endotoxemia, and higher levels of pro-inflammatory cytokine IL-6.^30^ Although data suggest that modifying the gut microbiome increases risk of infections, the extent to which opioid administration alone impacts these changes in humans has yet to be determined.^31^

It should be noted that one of the most common side effects of opioid use is constipation due to activation of MORs in the gastrointestinal tract.^13^ Constipation is known to be associated with alterations to the gut microbiota.^32^ There is some evidence to suggest that slowed intestinal transit leads to dysbiosis, creating a feed-forward loop whereby the altered microbiota further exacerbates constipation.^33^ Thus, it may be that some of the perturbations to the gut microbiota caused by opioids are a result of the impaired colonic motility.

### Differences in immunosuppression between opioids

The degree of immunosuppression appears to vary, depending on the type of opioid, duration of treatment, and dose used.^15,34^ Opioids that have been found to be more associated with immunosuppression include codeine, methadone, morphine, and remifentanil.^34^ Comparatively, buprenorphine, hydromorphone, oxycodone, and tramadol have been found to possess less immunosuppressive effects.^34^ Only morphine, fentanyl, and remifentanil have been found to be immunosuppressive in both animals and humans.^35^ Most evidence supporting the role of opioids in dampening the immune response comes from studies using morphine; however, morphine's effect on the immune system may not be generalizable to all opioids.^15,35^

Although it is not yet clear why there are differences in immunosuppressive properties of opioids, there are a few possibilities. Structural analysis has suggested an association between the chemical group at C_3_ and C_6_ and immunosuppression, as measured *in vitro* by lymphocyte proliferation, IL-2 production, and NK cell activity.^36^ Opioids with a hydroxyl group at C_3_ and C_6_ (morphine and nalorphine) had the strongest immunosuppressive effect, opioids with a C_3_ modification (codeine) were less immunosuppressive, and opioids with a carbonyl substitution at C_3_ (hydromorphone and oxycodone) lacked immunosuppressive effects.

It has been suggested that differences in immunosuppression are related to differences in receptor affinity based on the observation that opioids with low opioid receptor affinity tend to have lower immunosuppressive activity.^35^ However, this is only speculation and is not true of other opioids such as buprenorphine, which is not strongly immunosuppressive despite high binding affinity. Thus, this is not likely to be the sole factor influencing immunosuppression.

Another factor that may be at play is differential recruitment of signaling molecules. Each ligand has a different mechanism of binding, and in this way induce distinct conformational changes in the opioid receptor, which affects the formation of signaling complexes.^37,38^ For example, buprenorphine does not appear to recruit β-arrestin, or perhaps recruits it to a lesser extent, whereas morphine and methadone do.^35^ There is evidence that delta and kappa opioid receptors have immunomodulating downstream effects as well.^39^

In addition, crosstalk has been demonstrated between the opioid receptor and TLR-4, a key receptor that activates the immune response.^40^ Opioid receptor activation inhibits TLR-4 signaling, resulting in dampened immune responses. It may be that activity from these other receptors contributes to some of the variability of opioid immunosuppression.

## Clinical Evidence: Opioids and Increased Infection

In addition to the mechanistic and pathophysiological evidence of opioid-induced immunosuppressive effects on different levels of the immune system, there have been a multitude of epidemiological studies that have shown increased infection rates in patients taking opioids in acute and chronic settings. In a self-controlled case series of patients with rheumatoid arthritis, serious infections requiring hospital admission occurred 40% more often during periods of current opioid use compared with periods of nonuse.^41^

Another retrospective cohort study assessed the risk of serious infections, specifically among patients initiating use of long-acting opioids.^42^ In this study, hospitalizations due to infection were higher during periods of current use compared with past use and were highest in the first 30 days after opioid initiation. Of the individual opioids assessed (including morphine, fentanyl, methadone, oxycodone, and oxymorphone), only oxycodone had a significantly lower rate of infections compared with morphine.

Several observational studies have found increased risk of pneumonia associated with opioid use.^43–46^ In a nested case–control study of community-acquired pneumonia in older adults (aged 65–94 years), current opioid use increased risk of community-acquired pneumonia by 38% compared with nonuse.^44^ Risk of pneumonia was increased both with more recent (odds ratio [OR]: 3.24) and, to a lesser extent, chronic use (OR: 1.27). For those with chronic opioid use, risk of pneumonia varied by estimated daily dose with no clear pattern. The highest risk was seen in the 20 to 49 morphine milligram equivalents (MME) range.

A retrospective nested case–control study found current use of opioids was associated with 62% greater likelihood of invasive pneumococcal disease compared with controls after adjusting for known risk factors, including cardiovascular disease, serious hepatic disease, chronic lung disease, hemodialysis, and human immunodeficiency virus (HIV).^45^

A nested case–control analysis of the Veterans Aging Cohort Study used bivariable and multivariable conditional logistic regression analysis to analyze the risk of community-acquired pneumonia in people living with HIV.^46^ Both past (61–365 days before index date) and current use of opioids were associated with community-acquired pneumonia, even when excluding patients with an opioid use disorder. Risk tended to be greater among people living with HIV.

In all of these studies, greater risk of infection was seen with long-acting opioids versus short-acting, opioids classified by the authors as immunosuppressive versus nonimmunosuppressive and higher doses of opioids.^41,42,44–46^ Risk of infection decreased with longer time since opioid initiation in these studies.^44,45^

One of the biggest concerns from any infection is the potential for any infection to lead to sepsis, and subsequent organ/tissue damage and potential death. Thus, the evaluation of whether opioid use can increase susceptibility to sepsis is one of the most important outcomes to identify. Although studies in mice have shown increased susceptibility to sepsis in opioid-treated animals,^47,48^ there is a paucity of studies in humans.

One retrospective cohort study found a higher rate of sepsis in patients with opioid-related hospitalizations who died compared with patients with nonopioid-related hospitalizations who died (51.5% vs. 36.4%, *p* < 0.001).^49^ There is currently conflicting evidence on whether opioids worsen sepsis-related outcomes. One observational study investigated whether opioid-treated patients hospitalized for sepsis have increased mortality compared with nonopioid treated patients.^50^

Using electronic health records from 5994 patients with sepsis, the study found that opioid-treated patients had a significantly higher risk of death at 28 days, after adjusting for demographics, clinical comorbidities, severity of illness, and types of infection.^50^ In addition, the study found that gram-positive and gram-negative infections were significantly more common in opioid users versus nonusers (*p* < 0.001).^50^

Conversely, another study found significantly lower likelihood of in-hospital mortality in patients with sepsis among those with opioid-related hospitalizations compared with those with nonopioid-related hospitalizations, even after adjusting for baseline characteristics and severity of illness.^49^ However, it is important to note that this study evaluated opioid-related and nonrelated hospitalizations and did not evaluate all cases of opioid use compared with nonuse.

### Viral infections

In addition to bacterial infections, there is evidence that opioids have effects on the replication and pathogenesis of several viruses, including HIV, hepatitis C, influenza, respiratory syncytial virus, and herpes simplex virus (HSV).^51^ However, much of this evidence comes from *in vitro* or animal studies. Clinically, morphine administration in patients undergoing cesarean delivery is associated with reactivation of latent HSV,^51,52^ although clinical data supporting opioid-induced infection risk from the majority of known viruses is scarce.^51,52^

One of the more well-studied viruses in the context of opioid–pathogen interactions is HIV. Opioids are now considered cofactors in the immunopathogenesis of HIV, primarily due to their effect on chemokines and their receptors, which are important players in the pathogenesis of HIV.^53^ MOR agonists have been shown to amplify HIV replication *in vitro*.^53,54^ One study showed that when added to human fetal microglia and blood monocyte–derived macrophage cultures, methadone significantly enhanced HIV infection of these cells, with upregulation of CCR5 and enhanced viral activation and replication.^55^

In another preclinical study, morphine inhibited production of HIV-1-protective chemokines IL-8 and macrophage-inflammatory protein-1 and enhanced expression of the HIV-1 entry co-receptor genes CCR3, CCR5, and CXCR2.^56^ Studies in nonhuman primates infected with simian immunodeficiency virus have found that morphine administration exacerbated disease severity.^53,57,58^ It has been proposed that alterations to the gut microbiota leading to compromised intestinal barrier function, microbial translocation, and systemic inflammation is one factor contributing to disease progression in HIV-infected individuals.^59–61^

In a humanized mouse model of HIV, morphine promoted alterations to the gut microbiota, disruption of epithelial barrier integrity, and production of inflammatory cytokines.^62^ A similar profile of intestinal epithelial dysfunction was seen in opioid users with HIV.^62^ Thus, in addition to the ability of opioids to amplify HIV replication directly and enhance cellular infection of HIV itself, they may also contribute to HIV pathogenesis by exacerbating gut dysbiosis.

Although only a limited number of studies are available, there has been some evidence of an association between opioid use and COVID-19. Two independent meta-analyses have found significantly higher rates of hospitalization, admission to intensive care units, and mortality in opioid users compared with nonusers.^63,64^ However, further analysis is needed to elucidate the extent to which these findings were due to the immunosuppressive nature of opioids versus other effects of opioids that may increase susceptibility to worse outcomes with COVID-19, such as respiratory depression and end-organ damage.

### Risk of surgical site infections

Observational studies have consistently reported increased rates of infections in patients with both pre- and postoperative opioid use after total joint arthroplasty.^65–69^ One meta-analysis of three studies found preoperative opioid use (variably defined across studies, e.g., patients who met diagnostic criteria for opioid use disorder or any patient prescribed opioids in the previous six months) was associated with 36% greater risk of periprosthetic infection after total knee or hip arthroplasty.^68^

Another independent meta-analysis found 53% greater risk of periprosthetic joint infection after total hip arthroplasty with opioid use.^65^ A retrospective cohort study found a dose-dependent relationship between postoperative opioid use and risk of infectious complications.^67^ When adjusted for covariates, including preoperative opioid use, the adjusted ORs were as follows: 54–82 MME: 1.03 (95% confidence interval [CI]: 1.00–1.07); 83–116 MMEs: 1.11 (95% CI: 1.07–1.14); 117–172 MMEs: 1.14 (95% CI: 1.10–1.17); >172 MMEs: 1.37 (95% CI: 1.33–1.42).

Risk of infection due to opioid use also has been found for other types of surgeries. A 2020 retrospective study of patients receiving left ventricular assist device support reported increased incidence of sepsis among patients taking opioids (30%) versus patients not taking opioids (13%, *p* = 0.036) at one year.^70^ Another study found risk of superficial surgical site infection was almost three times greater in opioid users than nonopioid users after ventral and incisional hernia repair (adjusted OR: 2.9 [95% CI: 1.2–6.7]; *p* = 0.013).^71^ A multivariate analysis identified chronic opioid use as a risk factor for infection requiring operative wound washout in patients who underwent multilevel lumbar fusion (OR: 1.44; *p* = 0.025).^72^

### Risk of infections in patients with cancer

Up to 80% of patients with advanced cancer will require opioids for managing pain.^73^ It is difficult to assess whether opioid treatment is associated with increased susceptibility to infection in patients with cancer due to other causes of immunosuppression in these patients and other consequences of advanced illness. One of the first studies to investigate the relationship between opioids and infections in patients with cancer was a retrospective study published in 2013 by Suzuki et al.^74^

The study found that patients administered morphine had higher rates of infection than patients administered oxycodone (30% vs. 14%; OR: 3.6 [95% CI: 1.40–9.26] vs. 1.0), concluding that morphine was associated with higher infection rates in patients with cancer.^74^ Although the mean dose was higher in the morphine group, logistic regression analysis did not find a significant difference in risk of infection between those receiving higher (≥120 MME/day) and lower (<120 MME/day) doses.

Conversely, another retrospective study of stage IV cancer patients receiving palliative care did not observe differences in risk of infection between morphine and oxycodone. The authors noted, however, that the patients in their study were receiving a higher opioid dose and had more advanced illness than the patients in the study by Suzuki et al.,^74^ which may explain the discrepancy in findings. The study by Shao et al. found that rather than an association with any one opioid, the risk of developing infection increased by 2% per 10 mg increase in daily MME.^75^

However, it is possible that patients who require a higher opioid dose have greater underlying illness, making them more susceptible to infection. A 2019 study that used data from 2135 patients in the Cologne Cohort of Neutropenic Patients study did not observe an increased rate of infections with opioid use in patients with chemotherapy-induced neutropenia.^76^ The authors speculate that the short period of immunosuppression and inpatient setting may possibly explain the lack of association in their study. Thus, it is still unclear whether opioid treatment poses additional risk of infection to patients being treated for cancer-related pain.

### Limitations of the available evidence

The clinical studies that have assessed infection incidence in patients receiving opioids are observational and may be confounded by other risk factors for infection.^15^ For example, patients with greater opioid use may have been more prone to infection due to more advanced underlying disease or may express behaviors that confer a risk of infection.

Many of these studies employed multivariable analyses, which can help account for confounding variables, but still may not clearly separate risk due to immunosuppressive effects of opioids and other risk factors associated with opioid use. The current clinical evidence should be interpreted cautiously, and further prospective research is needed. Nonetheless, real-world studies provide valuable evidence to help inform the development of treatment paradigms. In the past decade, observational and real-world data have grown to be recognized as essential methods of clinical studies.^77–80^

## Opioid Antagonists to Ameliorate Opioid Immunosuppression

Considering the mounting evidence that opioids are immunosuppressive but remain important medications for relieving severe pain, research efforts are focused on reducing rates of infections in patient populations that require opioids in both the acute and chronic settings. One potential solution could be the use of opioid receptor antagonists such as naltrexone and naloxone, which may help reduce incidence of infections and improve outcomes.^81^ Naltrexone is primarily used as rehabilitation therapy for opioid use disorder to help prevent or reduce relapse, whereas naloxone is U.S. Food and Drug Administration approved to quickly reverse the effects of opioid overdose.^82,83^

Preclinical studies have demonstrated that naltrexone and naloxone can reverse the immunomodulatory effects of opioids, providing evidence that these effects are mediated by the MOR.^22,47,84,85^ *In vitro* studies have shown that naltrexone and naloxone can block opioid-induced inhibition of NK cell activity, antibody production, and macrophage phagocytosis and chemotaxis.^22^ One study in human immune cells latently infected with HIV showed that naltrexone blocked the morphine-mediated impairment of anti-HIV activity of CD8 T cells and had no effect on the CD8 T cells when administered alone.^84^

In animal experiments, these opioid receptor antagonists have been shown to reduce sepsis and mortality in opioid-treated animals after challenge with a pathogen.^22,47,85^ For example, one study examining the effect of morphine on susceptibility to *Salmonella typhimurium* infection in mice showed that none of the morphine-treated mice survived (compared with 46% survival in control-treated mice at 40 days) and they had 10^6^-fold greater bacterial burden compared with controls.^85^

When naltrexone was co-administered with morphine, median survival time increased from 3.2 days to 11.6 and bacterial growth was completely abrogated in the Peyer's Patches of 4 out of 5 mice. Again, naltrexone alone had no effect on survival or bacterial burden. Another study showed naltrexone protected against morphine-induced sepsis, reducing bacterial colonization of the liver, spleen, and peritoneal cavity compared with morphine alone.^47^ There is also evidence that opioid receptor antagonists can protect against opioid-induced microbiome changes. Naltrexone abolished morphine-induced changes to the gut microbiome and bile acid metabolism in mice.^26^

Clinical studies appear to corroborate preclinical data. In a recent randomized controlled trial, Krupitsky et al. showed that among patients with HIV and opioid use disorder, a two-month naltrexone implant reduced viral load at 48 weeks.^86^ As previously described, patients with opioid use disorder who were taking opioid agonists had reduced diversity of the gut microbiota.^29^ Patients receiving opioid antagonists (i.e., naloxone or naltrexone), whether alone or in combination with an agonist, showed similar diversity and richness as patients using neither opioid agonists nor antagonists.

However, nonselective opioid receptor antagonists such as naltrexone and naloxone completely negate the effects of opioid agonists and so cannot be used in patients who require ongoing opioid treatment for pain management or as medication-assisted treatment for opioid use disorder. In such patients, use of peripherally acting mu-opioid receptor antagonists (PAMORAs), which do not cross the blood–brain barrier, may be advantageous. These agents are unique in that they can help to ameliorate side effects without compromising opioid analgesia. Currently, methylnaltrexone, naldemedine, and naloxegol are PAMORAs indicated for the treatment of opioid-induced constipation (OIC).^87–89^

A limited number of studies are available on the ability of PAMORAs to ameliorate opioid-induced immunosuppression. In one *in vitro* study, pretreatment of human monocyte-derived macrophages with methylnaltrexone before incubation with morphine abrogated morphine-induced upregulation of the CCR5 receptor and HIV replication.^58^ The limited clinical data available so far indicate methylnaltrexone treatment can improve overall survival but have not yet assessed how this effect is mediated.

Methylnaltrexone is available as a tablet or injection for the treatment of adults with chronic noncancer pain and as an injection for adults with advanced illness or pain caused by active cancer.^90^ In a *post hoc* analysis of two randomized placebo-controlled clinical trials involving patients with advanced terminal cancers and OIC, subcutaneous administration of methylnaltrexone (0.15 or 0.3 mg/kg in one study, 8 or 12 mg based on body weight in the other)^91,92^ was associated with significantly longer overall survival compared with placebo (76 vs. 56 days, respectively; *p* = 0.033).^93^

Although it is possible that the improved survival could be due to improved gut function (as methylnaltrexone helped relieve OIC) or reversal of immunosuppression, it could also be due to anticancer activities of MOR antagonists. This may be supported by the finding that methylnaltrexone had no impact on survival in patients with advanced illness other than cancer. Another *post hoc* analysis assessed all-cause mortality in patients with noncancer pain or advanced illness who received subcutaneous, intravenous, or oral methylnaltrexone or matching placebo for OIC in 12 phase 2–4 randomized double-blind placebo-controlled studies.^94^

Methylnaltrexone reduced all-cause mortality by 60% compared with placebo (hazard ratio 0.399 [95% CI: 0.248–0.643]; *p* = 0.0002). This difference remained significant when adjusting for cancer status, age group, and gender (hazard ratio 0.508 [95% CI: 0.314–0.820]; *p* = 0.0056). However, these analyses were limited by an inability to conduct subgroup analyses with respect to cause of death due to relatively small sample size. It is not yet known whether other PAMORAs share this effect. One study of naldemedine in cancer patients with OIC found no significant difference in mortality between naldemedine- and placebo-treated patients.^95^

## Alternatives for Pain Management

Although use of opioid antagonists may be one way to mitigate the impact of opioids on the immune system, alternate pain management strategies may be necessary. Where possible, pain should be managed first by exercise and physical therapy. In patients that cannot be managed with a nonpharmacological approach, such as cancer patients, use of a less immunosuppressive opioid may be considered. Alternatively, opioids may be avoided altogether through use of approaches such as localized administration of steroids or anesthetic, radiofrequency ablation of nerves, transcutaneous electric nerve stimulation, peripheral nerve blocks, or spinal cord stimulation.^96^

## Conclusions

Observational clinical studies suggest that opioids are immunosuppressive, as demonstrated by increased infection rates with opioid use across various patient populations, even after adjusting for multiple confounders. The mechanism of immunosuppression involves direct and indirect effects and affects both innate and adaptive immunity. Substantial evidence from preclinical and clinical studies suggests that certain opioids are more immunosuppressive than others, which may be factors that can inform opioid choice in specific patients and patient populations.

Both selective and nonselective opioid receptor antagonists appear to partially block the immunosuppressive effects of opioids in early preclinical studies. Future studies are warranted to investigate whether the PAMORA methylnaltrexone is capable of reversing opioid-induced immunosuppression without compromising the analgesic effect of opioids; data to support this hypothesis are currently lacking. Given that most clinical evidence regarding the association between opioids and infections is observational, more prospective trials could help to corroborate the immunosuppressive effects of opioids in the clinical setting and to assess potential preventive treatments.

Both patients and caregivers should be educated about the potential risks of immunosuppression associated with opioid use. Future research is needed to understand the mechanisms underlying opioid-induced immunosuppression and find therapies that may be capable of reversing the immunosuppressive effects of opioids.

## Abbreviations Used

CI: confidence interval
CNS: central nervous system
COVID-19: coronavirus disease 2019
GPCR: G protein-coupled receptor
HIV: human immunodeficiency virus
HPA: hypothalamic–pituitary–adrenal
HSV: herpes simplex virus
IL: interleukin
MOR: mu-opioid receptor
NF-κB: nuclear factor-kappa B
NK: natural killer
OIC: opioid-induced constipation
OR: odds ratio
PAMORA: peripherally acting mu-opioid receptor antagonist
Th: helper T cell
TLR: toll-like receptors
